# Secondary Immunodeficiency and Vaccine Response in Rheumatoid and Psoriatic Arthritis on Immunosuppressive Medicines

**DOI:** 10.7759/cureus.66366

**Published:** 2024-08-07

**Authors:** Anthony J Ocon, Shiamak Cooper, Allison Ramsey, Shahzad Mustafa

**Affiliations:** 1 Rheumatology, Rochester Regional Health, Rochester, USA; 2 Rheumatology, University of Rochester Medical School, Rochester, USA; 3 Internal Medicine, Rochester Regional Health, Rochester, USA; 4 Allergy and Immunology, Rochester Regional Health, Rochester, USA; 5 Allergy and Immunology, University of Rochester Medical School, Rochester, USA

**Keywords:** psoriatic arthritis, biologic treatment, dmard therapy, rheumatoid arthritis, immunodeficiency syndrome

## Abstract

Background

Rheumatoid arthritis and psoriatic arthritis patients have dysregulated immune system parameters that may increase infection risk at baseline. In addition, treatment of these conditions with immunosuppressive medications may lead to the development of secondary immunodeficiency (SID). Our objective was to assess SID in a cohort on immunosuppressive medications. We hypothesized that SID is clinically detectable by assessing immune parameters and polysaccharide and protein-based vaccination responses.

Methodology

A prospective cohort study of 42 subjects on immunosuppressive medications was assessed. Analysis included immunoglobulin levels, lymphocyte subsets, and two-step response to diphtheria, tetanus, and 23-valent *Streptococcus pneumoniae* vaccinations. Exclusions included primary immunodeficiency, malignancy, pregnancy, neutropenia, immunoglobulin replacement, prior B-cell-depleting medication or chemotherapy, use of non-immunosuppressive medication, or recent use of glucocorticoids. Suboptimal vaccine response was defined as an abnormal response based on standard criteria for each vaccine.

Results

Low IgM levels (below 50 mg/dL) occurred in seven (17%) subjects and IgG (below 650 mg/dL) in three (7%) subjects. Impaired lymphocyte subsets were uncommon. In total, 33 (78%) subjects completed the two-step vaccination assessment. Overall, 29 of 33 (88%) subjects demonstrated suboptimal response to pneumococcal vaccination, 10 (30%) demonstrated suboptimal response to diphtheria, and four (12%) to tetanus. Two (6%) subjects demonstrated suboptimal response to all vaccinations. Finally, 31 (94%) subjects demonstrated suboptimal response to at least one vaccination.

Conclusions

SID may develop, is clinically detectable, and most notably demonstrated in suboptimal responses to polysaccharide vaccinations, especially against *S. pneumoniae*.

## Introduction

Rheumatoid arthritis (RA) and psoriatic arthritis (PsA) are systemic autoimmune diseases. They are among the most common causes of inflammatory arthritis, affecting millions of people worldwide. The immunology underlying both conditions is complex with dysregulation of cytokine production and immune cell responses [1,2]. Patients with these conditions may have an increased risk of infection at baseline due to immune system dysfunction. Those with higher disease activity, increased age, and additional comorbidities, such as diabetes, chronic kidney disease, or chronic lung disease, are especially at risk for infection [3]. The American College of Rheumatology recommends treatment with disease-modifying anti-rheumatic drugs (DMARDs), most of which are immunosuppressive [4,5]. Given the immunosuppressive nature of DMARDs, use may contribute to the development of secondary immunodeficiency (SID) [6-12], and infection related to medication use is a well-known complication.

Previously, Holmes et al. described that RA patients have an impaired response to pertussis vaccination, especially 5-10 years after vaccination, in female patients, and those using methotrexate [7]. Similarly, Kapetanovic et al. found that RA patients who use methotrexate have impaired antibody titer responses to the 23F and 6B polysaccharides of the 23-valent *Streptococcus pneumoniae* vaccine, but they did not look at the overall response to the other polysaccharide antigens or response to protein antigens [8]. They did not find that tumor necrosis factor (TNF) inhibitor use or prednisolone use affected titers. On the contrary, others suggest that TNF inhibition may inhibit the humoral response to the hepatitis B vaccination [9]. In PsA, there is less data on response to vaccinations. Mease et al. reported that the use of the TNF inhibitor etanercept did not affect the response to the 23-valent *S. pneumoniae* vaccine, but methotrexate did [13]. Chioato et al. reported that interleukin-17A inhibitor secukinumab did not affect response to influenza and meningococcal vaccinations in healthy subjects, but its response in PsA was not assessed [14]. Given that there are now multiple classes of DMARD medications used to treat RA and/or PsA, further assessment into the development of SID and impaired vaccine response concerning the use of these various medications is necessary.

The impact of the SARS-CoV-2 pandemic has demonstrated the need for effective vaccination as a public health response to mitigate serious diseases. However, rheumatologic patients, including those with RA or PsA, on DMARDs have blunted antibody responses to mRNA vaccinations and may require boosters for adequate efficacy [15-17]. Thus, it is in the public health interest of this high-risk cohort of patients to better understand their vaccine responses and whether they develop SID while on immunosuppressive medications.

Nonetheless, prior assessments evaluating SID in RA or PsA due to DMARD use are limited or incomplete due to only checking immunoglobulin levels or not assessing function response to both polysaccharide and protein-based vaccinations. Additionally, most studies do not control for concomitant use of glucocorticoids. Our objective was to fully assess SID in RA and PsA patients on DMARDs. We hypothesized that SID develops in RA and PsA patients on immunosuppressive medication and is identifiable by clinical immunologic assessment and suboptimal vaccine responses.

This data was presented in abstract form as a poster at the Rheumatology Research Workshop on May 16-17, 2024, in Denver, CO, USA.

## Materials and methods

Study subjects

Inclusion criteria included all individuals aged ≥18 years with a prior diagnosis of RA or PsA by a rheumatologist on immunosuppressive medication. Immunosuppressive medications could include conventional, biologic, and targeted synthetic DMARDs that were US Food and Drug Administration-approved for the treatment of RA or PsA. Dual therapy, including a combination of conventional DMARDs with other conventional, biologic, or targeted synthetic DMARDs, was allowed. All subjects were required to be on stable immunosuppressive therapy regimens for at least 90 days.

Exclusion criteria included prior immunosuppressive state (including primary immunodeficiency, malignancy, or neutropenia), current pregnancy or pregnancy in the prior 90 days, use of immunoglobulin replacement, an additional rheumatologic diagnosis, history of chemotherapy use, history of prior B-cell-depleting medication, and other immunosuppressive medication use. Given the well-known immunosuppressive effects, oral glucocorticoid medication over the previous 90 days was excluded. Use of apremilast or hydroxychloroquine as monotherapy was excluded. Combination therapy with hydroxychloroquine or aprelimast also was excluded. Prior joint surgery was allowed and not exclusionary.

Ethics and consent

The institutional review board of Rochester Regional Health reviewed and approved the study (approval number: 2150 B). All subjects signed written informed consent. The study was performed in accordance with the ethical standards and principles of the Declaration of Helsinki.

Study protocol

Upon consenting, subjects underwent serologic evaluations, including serum IgG, IgM, IgA, IgE, and lymphocyte subsets (CD3/4/8/19). Two-step vaccination titer assessment was done to determine appropriate vaccination responses [18]. First, baseline IgG titer responses to *S. pneumoniae*, *Corynebacterium diphtheriae*, and *Clostridium tetani* vaccinations were measured at enrollment. The subject then received the vaccinations (23-valent polysaccharide Pneumovax 23, Merck & Co., Inc., Rahway, NJ, USA; tetanus/diphtheria toxoids (Tenivac), Sanofi Pasteur Limited, Paris, France), and repeat titers were measured four weeks post-vaccination. IgG antibody titers were detected and semi-quantitated using the enzyme-linked immunosorbent assay methodology designed, validated, and performed by Mayo Clinic Laboratories (Mayo Clinic, Rochester, Minnesota, USA). For serum IgG, IgM, IgA, IgE, and lymphocyte subsets (CD3/4/8/19), low levels were defined as below the reference range minimum value.

Appropriate vaccination response was defined based on published criteria [18]. For *S. pneumoniae* (23-valent polysaccharide vaccine), an appropriate response was as follows: if titer <1.3, need to increase two-fold to above 1.3 or increase four-fold; or if titer >1.3, need to increase two-fold; and responses need to be demonstrated by 70% of serotypes. For diphtheria, an appropriate response was a two-fold increase in the protective range. For tetanus, an appropriate response was defined as a two-fold increase in the protective range. Suboptimal vaccine response was defined as an abnormal response to any of the above.

Data were collected prospectively. The study was conducted between March 22, 2022, through December 31, 2023. Data are presented as means with 95% confidence intervals (CIs) for continuous variables and numbers with percentages for discrete variables.

## Results

Demographics

A total of 42 subjects consented, of whom 26 (62%) had RA, and 16 (38%) had PsA. In total, 30 (71%) were female. Overall, 41 (98%) were Caucasian with one Hispanic subject. The mean age was 59.6 years (95% CI = 56.5-63.1 years). The mean time from diagnosis was 6.8 years (95% CI = 5.3-8.4 years). In total, 27 (64%) subjects were on monotherapy and 15 (36%) were on dual therapy. Table 1 shows the immunosuppressive medications used in the cohort.

**Table 1:** Immunosuppressant medication use. DMARD = disease-modifying anti-rheumatic drug; TNF = tumor necrosis factor; JAK = Janus kinase; RA = rheumatoid arthritis; PsA = psoriatic arthritis

Medication class	Number per diagnosis	Number per medication
Conventional DMARD	RA: 6; PsA: 3	Methotrexate: 3
		Leflunomide: 2
		Sulfasalazine: 4
TNF inhibitors	RA: 8; PsA: 3	Entanercept: 2
		Adalimumab: 9
Interleukin-6 inhibitors	RA: 2; PsA: n/a	Tocilizumab: 1
		Sarilumab: 1
Interleukin-17 inhibitors	RA: n/a; PsA: 3	Secukinumab: 2
		Ixekizumab: 1
T-cell costimulator inhibitor	RA: 1; PsA: 0	Abatacept: 1
JAK inhibitors	RA: 1; PsA: 0	Tofacitinib: 1
Dual therapy	RA: 8	Methotrexate/Tofacitinib: 1; Methotrexate/Adalimumab: 3; Methotrexate/Sulfasalazine: 1; Methotrexate/Leflunomide: 1; Methorexate/Abatacept: 1; Methotrexate/Golimumab: 1
	PsA: 7	Methotrexate/Etanercept: 1; Methotrexate/Adalimumab: 1; Methotrexate/Sulfasalazine: 1; Methotrexate/Secukinumab: 1; Leflunomide/Guselkumab: 1; Sulfasalazine/Secukinumab: 1; Sulfasalazine/Certolizumab: 1

Immunoglobulin levels and lymphocyte subsets

There were no low IgA or IgE values. Seven subjects (four RA and three PsA) (17%) had low IgM levels. Three subjects (two RA and one PsA) (7%) had low IgG levels. All three subjects with low IgG levels also had low IgM levels. Table 2 shows the ranges and medications used in subjects with low levels.

**Table 2:** Immunoglobulin and lymphocyte subset deficiencies. RA = rheumatoid arthritis; PsA = psoriatic arthritis

Value (reference range)	Number low per diagnosis	Values	Medication use (number of subjects)
IgM (50-300 mg/dL)	RA: 4	38, 40, 45, 48 mg/dL	Methotrexate (1), leflunomide (1), sarilumab (1), abatacept (1)
	PsA: 3	30, 33, 49 mg/dL	Methotrexate (1), sulfasalazine (1), ixekizumab (1)
IgG (650-1600 mg/dL)	RA: 2	570, 645 mg/dL	Leflunomide (1), abatacept (1)
	PsA: 1	564 mg/dL	Methotrexate (1)
CD3 (856-2669 cells)	RA: 3	702, 736, 787 cells	Methotrexate and tofacitinib (1), leflunomide (1), sarilumab (1)
	PsA: 0	-	-
CD4 (491-1734 cells)	RA: 1	388 cells	Methotrexate and tofacitinib (1)
	PsA: 0	-	-
CD8 (162-1074 cells)	RA: 1	67 cells	Methotrexate (1)
	PsA: 0	-	-
CD19 (73-562 cells)	RA: 1	71 cells	Methotrexate (1)
	PsA: 0	-	-

Lymphocyte subset deficiencies are also shown in Table 2. It was uncommon for RA patients to have deficiencies. No PsA patients had low CD3, CD4, CD8, or CD19 cell counts.

Diphtheria vaccination response

Overall, 33 of 42 (79%) subjects completed the two-step serologic vaccination assessment, with nine not completing it. In total, 23 of 33 (70%) subjects exhibited normal responses, and 10 of 33 (30%) exhibited suboptimal responses. Table 3 shows the medications used in subjects with suboptimal responses. One had low IgM and IgG levels and was on leflunomide.

**Table 3:** Suboptimal response to diphtheria or tetanus vaccine by medication.

	Number of patients
Diptheria vaccine inadequate response	
Methotrexate and tofacitinib	1
Leflunomide	1
Sulfasalazine	1
Etanercept	2
Adalimumab	2
Methotrexate and adalimumab	2
Sarilumab	1
Tetanus vaccine inadequate response	
Leflunomide	1
Adalimumab	1
Sarilumab	1
Ixekizumab	1

Tetanus vaccination response

Overall, 29 of 33 (88%) subjects exhibited normal responses, and four (12%) exhibited suboptimal responses. Table 3 shows the medications used in subjects with suboptimal responses. One had low IgM and IgG levels and was on leflunomide.

*Streptococcus pneumoniae* vaccination response

Four (12%) subjects exhibited normal responses, and 29 (88%) patients exhibited suboptimal responses. Table 4 shows the medications of those with a suboptimal response. Two had low IgM and three had low IgM and IgG.

**Table 4:** Suboptimal response to Streptococcus pneumoniae vaccine by medication DMARD = disease-modifying anti-rheumatic drug; TNF = tumor necrosis factor

Medication class	Number of patients
Conventional DMARDs	
Methotrexate	3
Leflunomide	1
Sulfasalazine	2
TNF inhibitor	
Etanercept	2
Adalimumab	6
Interleukin-6 inhibitor	
Sarilumab	1
Interleukin-17 inhibitor	
Secukinumab	2
Dual therapy	
Methotrexate and sulfasalazine	2
Methotrexate and leflunomide	1
Methotrexate and tofacitinib	1
Methotrexate and etanercept	1
Methotrexate and adalimumab	4
Methotrexate and golimumab	1
Sulfasalazine and secukinumab	1
Leflunomide and guselkumab	1
Total	29

Multiple suboptimal vaccination response

As shown in Table 5, two (6%) patients exhibited a suboptimal response to both diphtheria and tetanus vaccination; nine (27%) to both diphtheria and *S. pneumoniae* vaccinations; three (9%) to both tetanus and *S. pneumoniae* vaccinations; and two (6%) to all three vaccinations. Those two patients were on sarilumab or leflunomide. Only RA patients had suboptimal responses to more than one vaccine and no PsA patient did. Assessing for a suboptimal response to any vaccination, 31 (94%) subjects had at least one suboptimal response.

**Table 5:** Suboptimal vaccine response to more than one or any vaccination RA = rheumatoid arthritis; PsA = psoriatic arthritis

Multiple vaccination suboptimal response	N (%)	Disease
Diphtheria and tetanus	2 (6)	RA: 2; PsA: 0
Diphtheria and *S. pneumoniae*	9 (27)	RA: 9; PsA: 0
Tetanus and *S. pneumoniae*	3 (9)	RA:3; PsA: 0
All vaccinations	2 (6)	RA: 2; PsA: 0
Any vaccination	31 (94)	RA: 22, PsA: 9

## Discussion

Our data support the hypothesis that RA or PsA patients on immunosuppressive therapy may develop SID. SID was demonstrated by hypogammaglobinemia and suboptimal vaccination response. In our cohort, 94% of subjects exhibited at least one suboptimal vaccination response. Importantly, the response to the 23-valent *S. pneumoniae* vaccine was suboptimal in most subjects, with approximately one-third having a suboptimal response to diphtheria vaccination. This suggests that polysaccharide-based vaccination responses may be commonly impaired by immunosuppressant medications. The novelty of our study is emphasized in our assessing SID from a variety of diagnostic parameters, including immunoglobulin levels, lymphocyte subsets, as well as protein and polysaccharides vaccine responses, and excluding the use of confounding glucocorticoid medications.

Polysaccharide-based vaccines are less immunogenic than peptide-based vaccines. Polysaccharide vaccines typically induce a humoral B-cell response but are independent of a T-cell response [19]. The effectiveness of the 23-valent *S. pneumoniae* vaccine may vary in different populations but is still thought to be highly immunogenic [20-23], especially in a younger cohort of individuals, as included in our study. Our study suggests that immunosuppressive DMARD medications may impair the humoral response to this vaccine. Mechanistically, this is likely a heterogeneous process depending on the specific medication effect but may result in impaired B-cell clonal growth and antibody production [7,9,24,25], similar to specific antibody deficiency [26].

Given the heterogeneity of our cohort, it is difficult to draw conclusions about specific medications, but methotrexate and leflunomide were commonly associated with SID. Other studies have shown that methotrexate use may impair responses to influenza vaccination and mRNA SARS-CoV-2 vaccinations [16,27,28]. Vaccination data concerning leflunomide use is limited, but it may decrease response to SARS-CoV-2 mRNA vaccines [29,30]. Mechanisms behind this are likely multiple and include impaired cytokine signaling, impaired T-cell co-stimulation, and impaired B-cell differentiation, leading to decreased antibody production [31].

Clinically, assessment of SID should be considered in RA and PsA patients on immunosuppressive medications. In RA patients, an inherent dysregulation of the immune system, especially in those with increased disease activity, may increase infection risk [32]. Similarly, in patients with PsA compared to patients with only psoriasis, there is an increased risk for infection, again underlining a potential inherent immune dysregulation from the condition itself [33]. Therefore, SID due to medications in addition to disease-related immune dysregulation may be additive to infection risk. It is essential to try to ameliorate risk by administering appropriate vaccinations before the initiation of immunosuppressive medications, as well as to consider appropriate vaccine responses when possible. In those with suboptimal response, additional strategies for risk mitigation may be necessary, such as repeat vaccination or use of conjugated vaccines, as clinically warranted.

Our findings are in agreement with prior studies, but our study also builds on these prior findings. The use of abatacept with and without methotrexate in RA was reported to decrease pneumococcal vaccine responses, but this study did not assess all 23 serotypes, response to other vaccinations, or other immune parameters like our study did [34]. Methotrexate or methotrexate with a TNF inhibitor etanercept or infliximab in RA patients was shown to impair pneumococcal vaccine responses, but this study did not include other vaccinations, immune parameters, or assessment of other TNF inhibitors like our study did [8]. Moreover, the use of prednisolone was not excluded in that study, but our study controlled for this possible confounder. Previously, PsA patients on methotrexate were shown to have impaired pneumococcal vaccine responses, but etanercept use did not impair this [13]. This study did not look at the effects of other medications or immune parameters. Furthermore, JAK inhibitor baricitinib use with or without methotrexate in RA was shown to impair tetanus vaccine responses [12]. This study did not look at other immune parameters or other vaccinations. Overall, our study strengthens these prior findings by supporting them with additional immune parameter data as well as complete assessments of polysaccharide and protein-based vaccine responses.

Our main limitations were a small sample size and medication heterogeneity. We are, therefore, not able to conclude specific medications. Future prospective studies with larger cohorts for each individual medication class will be necessary to draw specific conclusions regarding medication class effects. Additionally, we did not correlate the development of SID with incident infections. Prospective studies assessing whether those who develop SID have a higher infection risk are warranted. As patients with RA or PsA often use various medications over their treatment course, we cannot determine the effect of prior medications. Finally, we did not have a control group of healthy subjects, nor did we have a group of untreated RA or PsA subjects for comparison given the ethical responsibility to treat subjects with these conditions. Thus, we cannot draw conclusions about specific effects on vaccination response which could intrinsically be related to having RA or PsA.

## Conclusions

We conclude that SID is prevalent in RA and PsA patients on immunosuppressive medications. Based on our findings, we suggest clinical evaluation of immunoglobulins and vaccine responses in this high-risk patient group. In particular, polysaccharide-based vaccination responses should be assessed. Patients with suboptimal responses may need risk mitigation strategies. This may include repeat vaccines or the use of conjugated vaccines. All forms of DMARD medications, including conventional, biologic, and targeted synthetic, may impart SID. Future large randomized controlled studies are needed to assess each particular class of medication for its impact on developing SID.
